# Naltrexone Treatment for Methamphetamine Dependence – Service Evaluation Audit

**DOI:** 10.1192/bjo.2024.517

**Published:** 2024-08-01

**Authors:** Emma Stimson, Owen Bowden-Jones

**Affiliations:** Central North West London NHS Foundation Trust, London, United Kingdom

## Abstract

**Aims:**

There is an emerging evidence base to support the benefit of naltrexone prescription in methamphetamine dependence. This audit assesses prescribing practice and benefit of naltrexone in a specialist NHS drug service based in West London. The process for initiation and monitoring of naltrexone in the service was compared with best practice recommendations.

A patient with methamphetamine dependence can be referred to a psychiatrist in order to consider naltrexone treatment. Naltrexone works by reducing cravings, thereby assisting with abstinence. Liver function is checked and then naltrexone is made available by an FP10 prescription. Follow up is then conducted in order to ascertain whether a continuation of naltrexone is indicated.

**Methods:**

Patients prescribed naltrexone were identified using a hand-written prescription record. Each case file was audited for prescribing metrics, substance misuse pattern, diagnoses, past treatments, efficacy, tolerability and length of prescription. Information was manually collected from the SystmOne case notes and anonymously entered into a spreadsheet under headed topics.

**Results:**

Data was collected from 1st April 2019 to 1st June 2023 which identified 28 patients. All patients had keyworker involvement and physical health checks. GHB/GBL was the most common comorbid substance. 18 of the 28 patients took naltrexone for longer than one week. 16 reported benefit with cravings. 6 were abstinent from methamphetamine and 10 were seen to have a partial response (periods of abstinence/lessened use). 9 of the 18 patients reported one or more side effects, most commonly nausea.

**Conclusion:**

The service meets best practice guidelines with regards to keyworker involvement, physical checks and follow-up reviews. Improvements could be made with regards to accurate diagnostic coding. Given the prevalence of side effects, it would be important to discuss options to mitigate these, as well as the importance of continuation of naltrexone (if tolerated) for at least four weeks. The offer of written information should be recorded. The tolerability and efficacy of naltrexone is in keeping with data from randomised controlled trials, which helps to inform patients and clinicians that naltrexone is an effective, safe treatment for methamphetamine dependence.

